# Effects of Catalase on Growth Performance, Antioxidant Capacity, Intestinal Morphology, and Microbial Composition in Yellow Broilers

**DOI:** 10.3389/fvets.2022.802051

**Published:** 2022-03-24

**Authors:** Minghong Tang, Rejun Fang, Junjing Xue, Kaili Yang, Yi Lu

**Affiliations:** ^1^College of Animal Science and Technology, Hunan Agricultural University, Changsha, China; ^2^Hunan Co-Innovation Center of Animal Production Safe (CICAPS), Changsha, China; ^3^Research and Development Center, Shanghai Menon Biotechnology Co., LTD, Shanghai, China

**Keywords:** catalase, yellow broilers, growth performance, intestinal morphology, antioxidant capacity, microbial composition, junction protein

## Abstract

The objective of this experiment was to study the effects of catalase (CAT) on growth performance, antioxidant capacity, intestinal morphology, and microbial composition of yellow broilers. Male Lingnan yellow broilers (360), aged 1 day, were randomly divided into control group (CON) (fed with a basic diet), R1 group (fed with basic diet + 150 U/kg catalase), and R2 group (fed with basic diet + 200 U/kg catalase). Each group had 8 replicates and 15 chickens in each replicate. The test is divided into the early stage (1–30 days) and the later stage (31–60 days). The results showed that compared with the control group, groups R1 and R2 significantly (*p* < 0.05) increased the weight gain and reduced (*p* < 0.05) the ratio of feed to gain in the early and the whole stages; prominently increased (*p* < 0.05) the concentration of total antioxidant capacity (T-AOC), the activities of CAT, superoxide dismutase (SOD), glutathione peroxidase (GSH-Px) in livers, the activities of CAT and GSH-Px in serum, and CAT in the jejunum in the early and the later stages; markedly increased (*p* < 0.05) the villus height and the ratio of villus height to crypt depth of the duodenum in the early and the later stages, the villus height and the villus height:crypt depth ratio of the jejunum and ileum in the early stage, and significantly lowered (*p* < 0.05) the crypt depth of the duodenum (in the early and the later stages), jejunum, and ileum (in early stage); memorably (*p* < 0.05) increased the number of total bacteria and Bacteroidetes in ceca, as well as the number of *Lactobacillus* in the jejunum (*p* < 0.05) on the 30th; significantly (*p* < 0.05) increased the mRNA expression of junction adhesion molecule 2 (JAM2), mucin 2 (MCU2), and occlusal protein (occludin) in the duodenum in the early stage, and increased (*p* < 0.05) the mRNA expression of JAM2 in the jejunum in the later stage. Collectively, adding catalase (CAT) to the diet of yellow broilers can improve the growth performance and the antioxidant capacity, promoting the integrity of intestinal morphology, optimizing the composition of intestinal microorganisms, and upregulating the mRNA expression of tight junction protein.

## Introduction

In the daily life activities of animals, free radicals (1) are constantly produced and cleared in the body. Under normal circumstances, the two processes maintain a dynamic balance (2, 3). However, when animals are affected by heat stress, disease, immunity, oxidized oil, mycotoxins, excessive metal ions, or other adverse factors (4–9), the balance between the production and clearance of free radicals is broken. The production of free radicals exceeds the ability and speed of body clearance, and the redox balance in the body is destroyed (10), causing the oxidative stress response of the body (11–15). Excess free radicals would attack biological macromolecules including DNA and protein, resulting in the peroxidation and dysfunction of biomacromolecules, thus, reducing the transcription level of genes and affecting the normal growth and development of the body. Growth performance, immune function, antioxidant function, intestinal microflora composition, intestinal morphology, and health status of animals are often affected and damaged (15–27).

The antioxidant system of animal is composed of the antioxidant enzyme system and non-antioxidant system (including various antioxidants), which can remove free radicals produced in the body (28–37). The antioxidant enzyme system is composed of CAT, SOD, GSH-Px, etc. CAT is a key enzyme in the antioxidant enzyme system, which has anti-inflammatory and antioxidant effects and widely exists in microorganisms, animals, and plants. It can catalyze the decomposition of hydrogen peroxide (H_2_O_2_), preventing iron chelates from using H_2_O_2_ and oxygen (O_2_) to generate more toxic hydroxyl radicals, preventing lipid oxidation of cell membrane, reducing oxidative damage.

The biological antioxidant system of yellow broilers is fragile due to rapid growth. Ambient high temperature, oxidized grease, high lipid, and protein diet (38), and high-density raising can cause oxidative stress easily (39). The scavenging capacity of free oxygen radicals and peroxides in the body decreases sharply, resulting in a large amount of accumulation (40–42), which affects the growth performance of the intestinal structure and body health of broilers. Therefore, this experiment was conducted to evaluate the effect of catalase (CAT) on growth performance, antioxidant capacity, intestinal morphology, microbial composition, and tight junction protein expression using 1-day-old male Lingnan yellow broilers as our research objective.

## Materials and Methods

### Catalase, Animals, and Diet

Catalase (CAT) was purchased from Shandong Longkete Enzyme Preparation Co., Ltd. (Shandong, China). The CAT is a food-grade enzyme preparation, and its enzyme activity is 5,000 U/g. Moreover, the definition of enzyme activity is at pH 7.0 and 30°C, decomposing 1 μmol H_2_O_2_ per minute; the amount of enzyme required is defined as 1 activity unit, expressed in U/g. The 1-day-old male Lingnan yellow broilers were purchased from the market chick seeding seller.

The basic diet was divided into the early stage (1–30 days) and the later stage (31–60 days). The diet was prepared in accordance with the standard National Research Council (43) and the industrial standard NY/T33-2004 of the People's Republic of China. The feed shape is powder, and the composition and nutritional level of the basic diet are listed in Table 1.

**Table 1:** Composition and nutrient levels of basal diets (air-dry basis).

**Ingredients (%)**	**Content**		**Nutrient compositions**	**Nutrient levels**	
	**Early stage** **(1 to 30 days)**	**Later stage** **(31 to 60 days)**		**Early stage** **(1 to 30 days)**	**Later stage** **(31 to 60 days)**
Corn	46.0	45.1	Metabolizable energy (MJ/kg)	12.55	13.39
Wheat	10.0	15.0	Crude protein (%)	21.00	19.0
Soybean meal (cp 43%)	32.5	25.1	Calcium (%)	0.90	0.85
Corn protein meal (cp 60%)	3.0	4.0	Available phosphorus (%)	0.45	0.42
Limestone	1.16	1.15	Lysine (%)	1.16	1.02
Dicalcium phosphate	1.67	1.55	Methionine (%)	0.5	0.46
Lard	3.85	6.25			
Sodium chloride	0.3	0.30			
*L*-Lysine HCl (98.5%)	0.36	0.40			
*DL*-Methionine (98%)	0.16	0.15			
Premix	1.00	1.0			

*The premix provided the following per kg of diets: VA 7,800 IU, VD_3_ 1,600 IU, VB_1_ 4.5 mg, VB_2_ 6.5 mg, VB_6_ 4.5 mg, VE 18 IU, VK_3_ 0.6 mg, VB_12_ 12 μg, nicotinic acid 38 mg, D-pantothenic acid 26 mg, folic acid 0.68 mg, biotin 0.3 mg, choline 650 mg; Cu 8 mg, Fe 80 mg, Zn 80 mg, Mn 60 mg, I 0.25 mg, Se 0.20 mg*.

*Nutrient levels were measured except for metabolizable energy (ME). ME was a calculated value*.

### Grouping and Management

A total of 360 healthy 1-day-old male Lingnan yellow broilers with similar weight were randomly divided into control group (CON) (fed basic diet), R1 group (fed basic diet + 150 U/kg CAT), and R2 group (fed basic diet + 200 U/kg CAT). There were 8 replicates in each group and 15 chickens in each replicate. The test period lasted for 60 days. The chickens were raised in three-layer cages and were immunized normally according to the feeding and management requirements of yellow broilers. Artificial light, free feeding, and clean drinking water were applied during the experiment period, and the daily behavior and health status of the chickens were observed in the experiment.

### Sample Collection and Processing

On the 30th and 60th days of the experiment, one chicken per replicate was selected for blood sample collection. The collected blood was centrifuged after standing, and the obtained serum was stored in the refrigerator at −20°C for subsequent testing.

After venous bloodletting to death, the bodies were immediately dissected, and the intestines were separated. The chyme of the jejunum and cecum was taken out in a sterile environment and stored at −80°C for total DNA extraction.

About 1 cm of duodenum, ileum, and jejunum were extracted, respectively, and gently washed away the contents with normal saline and fixed in 10% formalin solution. After dehydration, transparent treatment with xylene and paraffin embedding, the fixed intestinal segment was made into sections, which were stained and sealed for microscopic observation.

Intestinal segments of duodenum, jejunum, and ileum were cut out and washed with sterile phosphate buffer, then cut into pieces and stored at −80°C for RNA extraction.

Liver and jejunum tissues were washed off chyme with normal saline and placed in 2-ml cryopreservation tubes, respectively, and put in a −20°C refrigerator for analysis.

### Growth Performance

On the morning of the 1st, 30th, and 60th days of the experiment, the test chickens were weighed on an empty stomach (fasted for 12 h, only for drinking water). In addition, the daily feed intake and the number and weight of dead chickens were recorded to adjust the feed conversion ratio. Then the total feed intake and body weight on the 30th and 60th days of each repetition were counted, and the body weight gain, feed intake, and feed:gain ratio of each chicken at each stage were calculated as well.

### Antioxidant Indices

Total antioxidant capacity (T-AOC), catalase (CAT), superoxide dismutase (SOD), glutathione peroxidase (GSH-PX) activity, and malondialdehyde (MDA) content were detected in livers, serum, and jejunum using commercial assay kits, which were purchased from Nanjing Jiancheng Bioengineering Institute (Nanjing, China).

The thawed liver and jejunum samples were washed with precooled normal saline, dried with filter paper, and weighed. The weighed liver and jejunum samples were homogenized in the ice bath with precooled normal saline in a ratio of 1:9 by mass volume, centrifuged at 4°C for 20 min (rotating speed 10,000 r/min), and the supernatant was taken to determine the corresponding indices according to the xanthine oxidase method, colorimetry, spectrophotometry, and thiobarbital method.

### Intestinal Morphology

The prepared sections of the duodenum, ileum, and jejunum were photographed after selecting the typical field of vision with a fluorescence microscope, observed, and analyzed with Lecia Qwin image analysis system, and measured the villus height and crypt depth, respectively. Four visual field areas were taken from each section, and their average value was taken, and then the villus height:crypt depth was calculated (44).

### Microbial Composition

On the 30th day, 0.3 g each of thawed chyme of the jejunum and cecum were weighed, respectively, and the total DNA of the intestinal chyme was extracted by CTAB (cetyltrimethylammonium bromide), bead beating, and phenol–chloroform methods referring to the method of Zoetendal et al. (45). Simultaneously, using the kit (QIAamp Fast DNA Stool Mini Kit, Qiagen, Hilden, Germany), the total DNA from intestinal mucosa was extracted. With utilizing specific primers (Table 2), quantitative analysis of the total number of bacteria, Firmicutes, Bacteroidetes, *Clostridium* cluster IV, Clostridium cluster XIVα, *Lactobacillus*, and *Escherichia coli* were detected.

**Table 2:** Primers used for bacterial count real-time PCR.

**Target organisms**	**Sequences (5′-3′)**	**Product size/bp**	**References**
Total bacteria	F: GTGSTGCAYGGYYGTCGTCA R: ACGTCRTCCMCNCCTTCCTC	200	(46)
Firmicutes	F: GGAGYATGTGGTTTAATTCGAAGCA R: AGCTGACGACAACCATGCAC	126	(47)
Bacteroidea	F: GGARCATGTGGTTTAATTCGATGAT R: AGCTGACGACAACCATGCAG	126	(47)
*Clostridium* cluster IV	F: GCACAAGCAGTGGAGT R: CTTCCTCCGTTTTGTCAA	239	(48)
*Clostridium* cluster XIVα	F: CGGTACCTGACTAAGAAGC R: AGTTTYATTCTTGCGAACG	190	(48)
*Lactobacillus*	F: AGCAGTAGGGAATCTTCCA R: ATTCCACCGCTACACATG	345	(49)
*Escherichia coli*	F: CATGCCGCGTGTATGAAGAA R: CGGGTAACGTCAATGAGCAAA	95	(50)

### Relative Quantification of MRNA Expression of Tight Junction Protein

The 100 mg of duodenum, jejunum, and ileum tissues were weighed, homogenized by liquid nitrogen grinding method, and RNA was extracted by EASYspin Plus tissue/cell RNA rapid extraction kit (Beijing Adlai Biotechnology Co., Ltd. Beijing China).

The RNA concentration was determined by NanoDrop microspectrophotometer (nd-1000uv0vis, Thermo Fisher Scientific Inc.). Samples (cDNA>100 ng/μl) with OD260:OD280 values of 2.0–2.2 were stored at −20°C for reverse transcription. cDNA was synthesized by reverse transcription Kit (Prime Script TM RT reagent kit and gDNA Eraser kit, RR047a, Takara, Japan).

Gene quantitative PCR (qPCR) took cDNA as a template, and the primer sequence is shown in Table 3. The reaction system was: 10 μl f SYBR® Premix Ex Taq (TaKaRa Biotechnology, Dalian, China), 2 μl f DNA template, 0.3 μl each of upstream and downstream primers, and 8.4 μl of water. Quantification was performed using the CFX96 PCR System (Bio-Rad, USA). Quantitative results used glyceraldehyde-3-phosphate dehydrogenase (GADPH) as the internal reference gene. Each sample was repeated three times, and the gene expression was analyzed by the 2^−ΔΔCt^ method.

**Table 3:** Primers used for real-time PCR analysis.

**Target genes**	**Primer sequences (5′-3′)**	**Product size/bp**	**References**
*JAM*2	F: AGCCTCAAATGGGATTGGATT R: CATCAACTTGCATTCGCTTCA	59	(51)
*MUR2*	F: GCCTGCCCAGGAAATCAAG R: CGACAAGTTTGCTGGCACAT	59	(51)
Occludin	F: GAGCCCAGACTACCAAAGCAA R: ACCTCTGCCATCTCTCCACA	68	(51)
*ZO*-1	F: CCGCAGTCGTTCACGATCT R: GGAGAATGTCTGGAATGGTCTGA	63	(51)
*GAPDH*	F: GGCACGCCATCACTATC R: CCTGCATCTGCCCATTT	128	(52)

*JAM2, junction adhesion molecule 2; occludin, occlusal protein; ZO-1, cyclic protein-1; GADPH, glyceraldehyde-3-phosphate dehydrogenase*.

### Statistical Analysis

After the test data were processed by Excel 10, SPSS 19.0 statistical software was adopted for analysis of variance. When the difference was significant, Duncan's method was used for multiple comparisons. The test results were expressed as mean ± standard deviation, and *p* < 0.05 was significant.

## Results

### Growth Performance

As Table 4 shows, there was a significant difference in body weight gain and feed:gain ratio among the three test groups from 1 to 30 days (*p* < 0.05), and compared with the control group and R2 group, the R1 group increased feed intake significantly (*p* < 0.05). From 31 to 60 days, the body weight gain of the three experimental groups showed an upward trend, but there was no remarkable difference (*p* > 0.05); however, groups R1 and R2 decreased the ratio of feed to gain significantly (*p* < 0.05) compared with the control group in this stage. In the whole course (from 1 to 60 days), body weight gain and feed:gain ratio of R1 and R2 groups were significantly improved (*p* < 0.05) than those of the control group. Simultaneously, there was a significant difference in feed intake between the group R1 and the control group (*p* < 0.05).

**Table 4:** Effects of catalase (CAT) on growth performance of yellow broilers.

**Items**		**Weight gain/g**	**Feed intake/g**	**Feed: Gain**
1–	CON	499.20 ± 12.31^c^	1011.42 ± 16.17^b^	2.03 ± 0.05^a^
30 days	R1	559.12 ± 8.45^b^	1046.17 ± 15.91^a^	1.87 ± 0.02^b^
	R2	585.73 ± 14.60^a^	1019.43 ± 11.29^b^	1.74 ± 0.05^c^
31–	CON	729.34 ± 61.58	2034.35 ± 40.28	2.81 ± 0.22^a^
60 days	R1	783.58 ± 76.02	2056.45 ± 33.87	2.65 ± 0.25^b^
	R2	781.15 ± 67.82	2043.47 ± 36.26	2.63 ± 0.18^b^
1–	CON	1228.54 ± 68.37^b^	3045.77 ± 50.32^b^	2.48 ± 0.12^a^
60 days	R1	1342.71 ± 76.50^a^	3102.62 ± 32.80^a^	2.35 ± 0.14^b^
	R2	1366.88 ± 67.01^a^	3062.90 ± 40.00^ab^	2.24 ± 0.08^b^

*Data are the mean ± SEM (n = 8). CON-broilers fed the basal diet, R1-broilers fed the basal diet supplemented with 150 U/kg of CAT, R2-broilers fed the basal diet supplemented with 200 U/kg of CAT*.

a,b,c *Values within the same period and the same column with different letters differ (p < 0.05)*.

### Antioxidant Indices

The data on antioxidant indices are summarized in Table 5. On the 30th day, the activities of CAT in jejunums showed a significant increase (*p* < 0.05) among the three test groups; compared with the control group, adding 150 and 200 U CAT to the diet significantly increased (*p* < 0.05) the concentration of T-AOC, the activities of CAT, SOD, and GSH-Px in the liver, and the activities of CAT and GSH-Px in serum. In the meantime, dietary supplementation with 200 U CAT displayed a notable decrease (*p* < 0.05) in the content of MDA in the serum and jejunum. On the 60th day, groups R1 (150 U CAT) and R2 (200 U CAT) increased (*p* < 0.05) the concentration of T-AOC, the activities of CAT, SOD, and GSH-Px in the liver, the concentration of T-AOC and the activity of GSH-Px in the serum, and the activity of CAT in jejunums. CAT activity in the serum increased prominently among the three groups (*p* < 0.05).

**Table 5:** Effects of CAT on antioxidant indices of yellow broilers.

**Items**			**T-AOC**	**CAT**	**SOD**	**GSH-Px**	**MDA**
30th day		**The unit of measure**	**mmol/g prot**	**U/g prot**	**U/mg prot**	**U/g prot**	**nmol/mg prot**
	Liver	CON	0.12 ± 0.02^b^	19.59 ± 6.23^a^	941.63 ± 189.63^b^	33.22 ± 18.79^b^	0.42 ± 0.12
		R1	0.18 ± 0.02^a^	53.58 ± 15.00^b^	1,182.80 ± 181.60^a^	64.90 ± 13.96^a^	0.36 ± 0.15
		R2	0.21 ± 0.05^a^	66.60 ± 18.87^b^	1,234.62 ± 422.53^a^	66.29 ± 22.55^a^	0.33 ± 0.12
		**The unit of measure**	**U/ml**	**U/ml**	**U/ml**	**U/ml**	**nmol/ml**
	Serum	CON	11.65 ± 1.86	3.44 ± 0.80^b^	121.80 ± 13.83	570.36 ± 73.19^b^	3.29 ± 0.58^a^
		R1	13.13 ± 1.75	4.43 ± 0.60^a^	135.03 ± 16.14	695.69 ± 87.93^a^	3.10 ± 0.52^ab^
		R2	13.50 ± 3.11	4.82 ± 0.72^a^	139.13 ± 25.98	715.43 ± 125.54^a^	2.72 ± 0.30^b^
		**The unit of measure**	**mmol/g prot**	**U/g prot**	**U/mg prot**	**U/g prot**	**nmol/mg prot**
	Jejunum	CON	0.11 ± 0.02	5.68 ± 2.37^c^	621.84 ± 63.73	71.87 ± 25.70	2.23 ± 1.70^a^
		R1	0.12 ± 0.03	16.63 ± 7.97^b^	680.65 ± 140.05	100.03 ± 44.63	1.82 ± 1.31^ab^
		R2	0.12 ± 0.03	23.22 ± 4.48^a^	696.81 ± 90.19	103.76 ± 33.04	1.65 ± 0.96^b^
60th day		**The unit of measure**	**mmol/g prot**	**U/g prot**	**U/mg prot**	**U/g prot**	**nmol/mg prot**
	Liver	CON	0.11 ± 0.02^b^	33.55 ± 21.07^b^	1,056.54 ± 212.78^b^	26.50 ± 14.87^b^	0.36 ± 0.14
		R1	0.15 ± 0.05^a^	69.29 ± 54.80^a^	1,269.69 ± 180.37^a^	53.56 ± 14.24^a^	0.30 ± 0.13
		R2	0.17 ± 0.02^a^	76.91 ± 45.35^a^	1,303.23 ± 193.14^a^	54.56 ± 15.32^a^	0.26 ± 0.09
		**The unit of measure**	**U/ml**	**U/ml**	**U/ml**	**U/ml**	**nmol/ml**
	Serum	CON	13.04 ± 1.55^b^	4.53 ± 1.03^c^	167.24 ± 13.85	678.52 ± 110.56^b^	2.53 ± 0.54
		R1	17.00 ± 2.24^a^	6.29 ± 0.73^b^	179.54 ± 18.75	869.29 ± 139.07^a^	2.45 ± 0.34
		R2	17.54 ± 1.50^a^	8.07 ± 1.79^a^	184.16 ± 17.86	1,005.47 ± 143.97^a^	2.40 ± 0.34
		**The unit of measure**	**mmol/g prot**	**U/g prot**	**U/mg prot**	**U/g prot**	**nmol/mg prot**
	Jejunum	CON	0.10 ± 0.03	5.25 ± 2.20^b^	590.41 ± 60.51	68.55 ± 20.21	2.13 ± 1.59
		R1	0.11 ± 0.02	16.00 ± 7.07^a^	626.38 ± 106.40	92.92 ± 38.89	1.77 ± 1.27
		R2	0.12 ± 0.03	20.74 ± 3.29^a^	643.46 ± 125.67	97.29 ± 30.46	1.72 ± 1.30

*Data are the mean ± SEM (n = 8). CON-broilers fed the basal diet, R1-broilers fed the basal diet supplemented with 150 U/kg CAT, R2-broilers fed the basal diet supplemented with 200 U/kg of CAT*.

a,b,c *Values within the same period and the same column with different letters differ (p < 0.05). T-AOC, total antioxidant capacity; SOD, superoxide dismutase; GSH-Px, glutathione peroxidase; MDA, malondialdehyde*.

### Intestinal Morphology

Data in Table 6 shows that on the 30th day, compared with the control group, the addition of 150 and 200 U CAT in the diet could markedly (*p* < 0.05) increase the villus height and villus height:crypt depth ratio of the duodenum, jejunum, and ileum, and significantly reduce (*p* < 0.05) crypt depth. On the 60th day, in comparison with the control group, the villus height and the ratio of villus height to crypt depth of the duodenum and jejunum were improved notably (*p* < 0.05) after adding 150 and 200 U CAT (*p* < 0.05). Dietary supplementation with 200 U of CAT notably lowered (*p* < 0.05) crypt depth of the ileum and significantly increased (*p* < 0.05) the ratio of villus height to crypt depth. At the same time, there were conspicuous differences in the crypt depth of the duodenum among the three groups (*p* < 0.05).

**Table 6:** Effects of CAT on intestinal morphology of yellow broilers.

**Items**	**Duodenum**			**Jejunum**			**Ileum**		
	**Villous height/μm**	**Crypt depth/μm**	**Villous height/crypt depth**	**Villous height/μm**	**Crypt depth/μm**	**Villous height/crypt depth**	**Villous height/μm**	**Crypt depth/μm**	**Villous height/crypt depth**
**30d**									
CON	1,078.73 ± 173.30^b^	148.76 ± 28.17^a^	7.46 ± 1.72^b^	814.71 ± 41.87^b^	135.79 ± 25.58^a^	6.19 ± 1.21^b^	620.03 ± 41.26^b^	121.00 ± 17.85^a^	5.21 ± 0.74^b^
R1	1,259.76 ± 136.30^a^	106.53 ± 16.41^b^	12.1 ± 2.44^a^	969.73 ± 58.93^a^	93.21 ± 9.92^b^	10.06 ± 0.75^a^	707.88 ± 32.53^a^	91.15 ± 24.69^b^	8.20 ± 1.95^a^
R2	1,301.29 ± 65.09^a^	104.66 ± 18.56^b^	12.77 ± 2.33^a^	933.25 ± 65.53^a^	85.14 ± 21.27^b^	12.33 ± 4.44^a^	719.67 ± 33.07^a^	83.20 ± 15.14^b^	8.91 ± 1.70^a^
**60 days**									
CON	1,409.31 ± 83.85^a^	211.68 ± 28.28^a^	6.74 ± 0.80^c^	758.50 ± 42.89^b^	164.95 ± 43.08	4.89 ± 1.33^b^	832.47 ± 77.48	141.07 ± 32.12^a^	6.21 ± 1.60^b^
R1	1,587.57 ± 115.14^b^	175.50 ± 47.54^b^	9.60 ± 2.39^a^	1,086.34 ± 42.56^a^	156.06 ± 17.06	7.04 ± 0.84^a^	879.54 ± 38.84	133.21 ± 37.30^ab^	7.29 ± 2.90^ab^
R2	1,542.84 ± 103.74^b^	121.91 ± 16.20^c^	12.79 ± 1.36^a^	1,098.19 ± 70.71^a^	150.31 ± 20.54	7.48 ± 1.44^a^	863.99 ± 32.06	102.13 ± 21.54^b^	8.80 ± 1.88^a^

*Data are the mean ± SEM (n = 8). CON-broilers fed the basal diet, R1-broilers fed the basal diet supplemented with 150 U/kg of CAT, R2-broilers fed the basal diet supplemented with 200 U/kg of CAT*.

a,b,c *Values within the same period and the same column with different letters differ (p < 0.05)*.

### Microbial Composition

**Figure 1:**
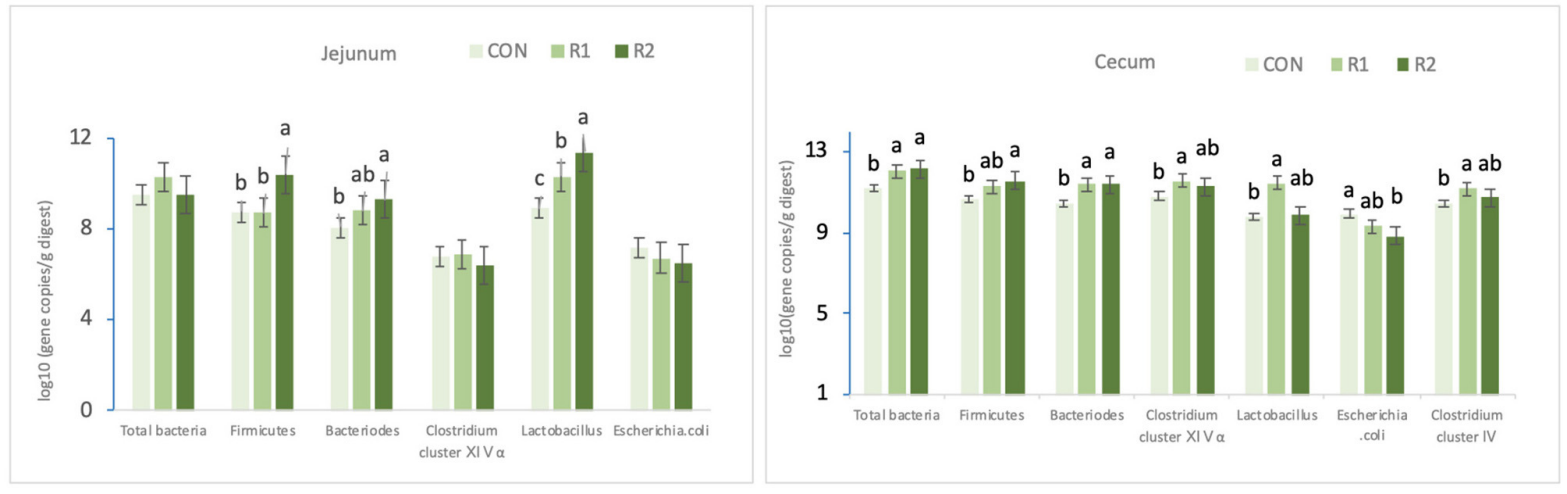
Effects of dietary catalase supplementation on intestinal microbial composition of 30-day-old yellow broilers. Data are the mean ± SEM (*n* = 8). CON-broilers fed the basal diet, R1-broilers fed the basal diet supplemented with 150U/kg CAT, R2-broilers fed the basal diet supplemented with 200U/kg CAT^a,b,c^ within the same intestinal segment and the same strain, means with different superscript letters differ (*P* < 0.05).

According to the data in Table 7 and Figure 1, on the 30th day, the diet supplemented with 200 U of CAT prominently increased (*p* < 0.05) the jejunal Firmicutes and Bacteroidetes counts and significantly decreased the number of *Escherichia coli* in the ceca (*p* < 0.05). Adding 150 and 200 U of CAT could remarkably increase (<0.05) the number of Bacteroidetes and total bacteria in the ceca; diet with the 150 U of CAT could effectively increase (*p* < 0.05) the amount of *Clostridium* cluster IV, *Clostridium* cluster XIVα, and *Lactobacillus* counts in the ceca. Also, the quantity of *Lactobacillus* in the jejunum increased markedly among the three groups (*p* < 0.05).

**Table 7:** Effects of CAT on the intestinal microbial composition of 30-day old yellow broilers.

**Items**	**Jejunum**			**Cecum**		
	**CON**	**R1**	**R2**	**CON**	**R1**	**R2**
Total bacteria	9.50 ± 0.59	10.25 ± 0.1.11	9.52 ± 3.45	11.20 ± 0.24^b^	12.06 ± 0.54^a^	12.16 ± 0.69^a^
Firmicutes	8.75 ± 0.39^b^	8.70 ± 1.23^b^	10.40 ± 0.59^a^	10.65 ± 0.34^b^	11.28 ± 0.62^ab^	11.56 ± 0.80^a^
Bacteroidetes	8.06 ± 6.74^b^	8.78 ± 1.25^ab^	9.28 ± 1.20^a^	10.47 ± 0.55^b^	11.37 ± 0.45^a^	11.38 ± 0.69^a^
*Clostridium* cluster IV	–	–	–	10.46 ± 0.28^b^	11.17 ± 0.26^a^	10.75 ± 0.63^ab^
*Clostridium* cluster XIVα	6.75 ± 0.50	6.90 ± 0.39	6.42 ± 0.61	10.81 ± 0.53^b^	11.58 ± 0.48^a^	11.28 ± 0.70^ab^
Lactobacillus	8.90 ± 0.51^c^	10.30 ± 1.04^b^	11.37 ± 0.32^a^	9.81 ± 0.57^b^	11.44 ± 0.77^a^	9.85 ± 1.14^ab^
*Escherichia coli*	7.21 ± 0.49	6.73 ± 0.76	6.49 ± 0.99	9.93 ± 0.70^a^	9.32 ± 1.15^ab^	8.82 ± 0.94^b^

*Data are the mean ± SEM (n = 8). CON-broilers fed the basal diet, R1-broilers fed the basal diet supplemented with 150 U/kg of CAT, R2-broilers fed the basal diet supplemented with 200 U/kg of CAT*.

a,b,c *Values within the same intestinal segment and the same row with different letters differ (p < 0.05)*.

### mRNA Expression of Tight Junction Protein

**Figure 2:**
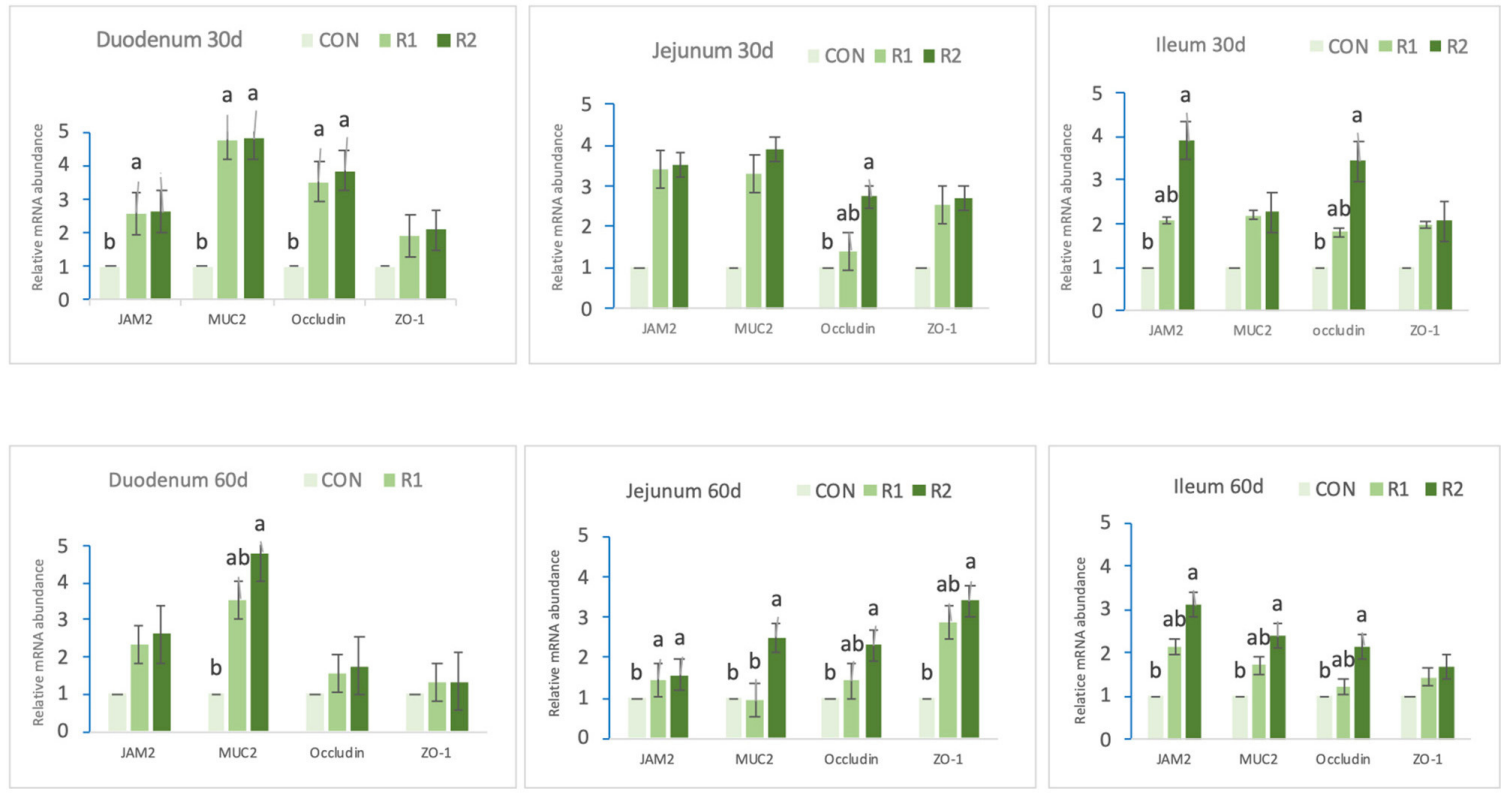
Effects of dietary catalase supply on tight junction protein mRNA expression in the intestinal mucosa of yellow broilers. Data are the mean ± SEM (*n* = 8). CON-broilers fed the basal diet, R1-broilers fed the basal diet supplemented with 150 U/kg CAT, R2-broilers fed the basal diet supplemented with 200 U/kg CAT^a,b,c^ within the same intestinal segment and the same protein, means with different superscript letters differ (*p* < 0.05).

The results obtained from the mRNA expression of tight junction protein are presented in Figure 2. On the 30th day, compared with the control group, group R1 (150 U CAT) and R2 (200 U CAT) increased (*p* < 0.05) the expression levels of JAM2, MUR2, and occludin in the duodenum. Additionally, group R2 (200 U CAT) significantly upregulated (*p* < 0.05) jejunal, occludin, ileal JAM2, and occludin mRNA expression. On the 60th day, compared with the control group, group R2 (200 U CAT) increased notably (*p* < 0.05) the expressions of duodenal MUR2, jejunal MUR2, occludin, and closed small cyclic protein-1 (ZO-1), ileal JAM2, MUR2, and Occludin. In the meantime, groups R1 and R2 dramatically heightened (*p* < 0.05) the expression level of jejunal JAM2.

## Discussion

Rapid growth, high lipid and protein feed, and high-density cage feeding are high-intensity stresses for broilers. These stresses (53) can lead to the production of a large number of free radicals, break the steady-state balance of free radical production and clearance in the body, and then induce oxidative stress in the digestive tract, damage the intestinal mucosa (54–56), and affect the enzyme activity. As a result, the digestion and absorption capacity of the digestive tract to feed decreased (57, 58), showing that the growth of broilers slowed down, and the feed:gain ratio increased (59, 60). CAT, as a very important enzyme in the antioxidant enzyme system, can quickly decompose H_2_O_2_ and eliminate the harm caused by H_2_O_2_. The active unpaired electrons in the outer orbit of free radicals can be transferred between different atoms or ions, so the free radicals can be transformed with each other in a chain reaction (61). At the same time, CAT, SOD, and GSH-Px have synergistic effects on free radical scavenging. Therefore, CAT can remove free radicals in the intestine by continuously decomposing H_2_O_2_, attenuate or eliminate intestinal oxidative stress, ameliorate the structure of intestinal mucosa, improve the digestion and absorption capacity of nutrients (62–66), promote the growth of broilers, and abate the F:G ratio. The results showed that the addition of 150 and 200 U of CAT in the diet could significantly enhance the body weight gain of yellow broilers in the early and the whole stages, and dramatically reduce the ratio of feed:gain in the early, the later, and the whole stages.

In the life process of animals, free radicals widely exist in the body. Low-dose free radicals can act as signal transduction molecules (67, 68), mediate biological defense (69, 70), regulate the expression of antioxidant enzymes in the body, and maintain the internal balance of redox (67, 71). However, a high concentration of free radicals will reduce the activity of antioxidant enzymes, do harm to the body, and threaten the growth, development, and health of animals (72). CAT, SOD, and GSH-Px together constitute the antioxidant enzyme system of the body, and they work together to clean up free radicals (73, 74) and maintain the balance of free radical production and elimination. The addition of CAT to the diet cannot only remove free radicals, reduce the concentration of free radicals, decrease the production of MDA in the body, and save the consumption of other antioxidant enzymes but also induce the expression of CAT, SOD, and GSH-Px in the body (67, 75), and improve the antioxidant capacity of the body. As expected, the results displayed that adding 150 and 200 U of CAT to the diet could significantly increase the T-AOC concentration, SOD, CAT, and GSH-Px activities in the liver, CAT and GSH-Px activities in serum and jejunal CAT activities in the early stage, and remarkably heighten the T-AOC concentration, SOD, CAT, and GSH-Px activities in the liver, T-AOC concentration, CAT and GSH-Px activities in the serum, and jejunal CAT activities in the later stage. Furthermore, the addition of 200 U of CAT significantly decreased the content of MDA in the serum and jejunum in the early stage.

Endogenous or exogenous stress (53) causes the production of excess free radicals in the intestine (76), which triggers oxidative stress in the digestive tract, leads to the apoptosis and abscission of intestinal mucosal cells (77), and the differentiation and maturation of crypt intestinal stem cells are destroyed. Therefore, after oxidative damage, intestinal mucosal villi atrophy and fall off, villus height decreases, and crypt depth increases (58). Brollegier et al. found that heat stress markedly reduced the height, volume, and surface area of jejunal villi in young hens. The addition of CAT in the diet can remove excessive free radicals in the intestine, dispel or weaken intestinal oxidative stress, return to the normal differentiation and maturation of intestinal stem cells, and repair the injured intestinal mucosa, improving the height of villi and debasing the depth of the recess.

The study results indicated that diet supplemented with 150 and 200 U of CAT can significantly increase the villus height and villus height:crypt depth ratio of the duodenum (in the early and the later stages), jejunum (in the early stage), and ilea (in the early stage), as well as significantly reduce (*p* < 0.05) crypt depth. The addition of 200 U of CAT prominently lowered crypt depth and dramatically raised the ratio of villus height to crypt depth of the ileum in the later stage.

The intestinal microorganisms are divided into mucosal microorganisms and intestinal microorganisms (78). The mucosal microorganisms mainly colonize the mucus layer of intestinal epithelial cells. Meanwhile, the intestinal microorganisms exist in the intestinal cavity. The intestinal microorganisms continuously plant in the intestinal epithelium, and the mucosal microorganisms continuously inoculate into the intestinal cavity. Under healthy conditions, this state maintains a dynamic balance (79, 80). When the intestinal tract is subjected to oxidative stress of free radicals, the balance is broken and changes the composition of intestinal microorganisms (81, 82). The influence of intestinal oxidative stress on intestinal microorganisms is mainly caused by the following reasons: ① Oxidative stress leads to intestinal mucosal damage, intestinal epithelial cell apoptosis, destroys the colonization basis of mucosal microorganisms, and affects the proliferation of microorganisms. ② After the intestinal mucosa is damaged, the permeability of the intestinal mucosa is improved, the barrier function of the intestinal mucosa is reduced, and the migration of microorganisms inside and outside the intestinal mucosa becomes easier, thus, affecting the composition of microorganisms. ③ After the intestinal mucosa is damaged, the osmotic pressure and permeability are increased (83), mucus release and electrolyte secretion occur (57), which changes the pH in the intestine and breaks the microenvironment for the growth of intestinal microorganisms. ④ The free radicals produced by oxidative stress directly attack the biofilm of intestinal microorganisms (84), causing harm to the normal life activities of microorganisms. There are great differences in the antioxidant enzyme systems of different intestinal microorganisms, so their tolerance to oxidative stress is different. After intestinal oxidative stress, the number of different microorganisms changes greatly. A number of studies have shown that dietary supplementation with essential oil, probiotics, acidifier, and iron oxide nanozyme can inhibit the growth of harmful bacteria, reduce their infection ability, increase the number of beneficial bacteria and consolidate their dominant position, and alleviate the effects of stress on intestinal microorganisms. The addition of CAT can eliminate free radicals in the intestine, alleviate oxidative stress, reduce the damage of intestinal mucosa, and regulate the composition of intestinal microorganisms (85–87).

In the current study, adding 150 U of CAT could significantly increase the cecal *Clostridium* cluster IV, *Clostridium* cluster XIVα, and *Lactobacillus* amounts. The addition of 200 U of CAT could prominently heighten the number of Firmicutes, Bacteroidetes in the jejunum and Firmicutes in the ceca, and markedly reduce the number of *Escherichia coli* in the ceca. On the other hand, dietary supplementation with 150 and 200 U of CAT could significantly enhance the quantity of *Lactobacillus* in the jejunum and total bacteria, Bacteroidetes in the ceca.

The tight junction is the main connection mode between intestinal epithelial cells (88), which is composed of the transmembrane protein and plaque protein, regulates the permeability of epithelial cells, and mediates material exchange and energy metabolism (89). Transmembrane proteins consist of occludin, claudin, and adhesion molecule (JAM), and plaque proteins are mainly formed of zonula (ZO) family proteins (90). Occludin forms the occludin–ZO complex on the outside of the cell membrane and plays its physiological role. Occludin and ZO-1 are the two most important tight junction proteins (91). Previous studies have shown that the decreased expression of claudin and ZO-1 in the intestinal tissue will increase intestinal permeability, and the intestinal barrier function could be compromised (92, 93). JAM2, as an important adhesion molecule, binds to tight junction proteins such as ZO-1 through a special domain (PDZ domain). Therefore, regulating the expression of JAM2 can promote and enlarge the generated quantity of tight junction proteins (94, 95) and enhance the barrier function of the intestinal mucosa. Mucus covering the surface of the intestinal epithelial cells is the macromolecular glycoprotein secreted by goblet cells, forming a mucus barrier and protecting intestinal mucosa (96, 97). MUR2 is an important component of mucin and a marker gene to judge the integrity of the intestinal mucus barrier (98). The debased expression of MUR2 affects and destroys the barrier function of mucus (99). Free radicals produced by oxidative and disease stress attack intestinal epithelial cells, resulting in DNA, protein, and lipid peroxidation (100–105), lessen the expression of occludin, ZO-1, JAM2, and MUR2 (106, 107), improve the permeability of intestinal epithelial cells, and weaken the barrier function (92, 93, 108, 109).

Assimakopoulos et al. (110) found that obstructive jaundice markedly downregulated the expression of intestinal occludin. Yang et al. (111) confirmed that obstructive jaundice significantly reduced the expression of occludin and ZO. Forder et al. (112) reported that the mRNA expression of MUR2 in the jejunum decreased remarkably after *Eimeria* and *Clostridium perfringens* double infection in broilers. When CAT was added to the diet to rapidly scavenge free radicals and weaken intestinal oxidative stress injury, the expression of a series of proteins such as occludin, ZO-1, JAM2, and MUR2 increased.

Results of this study indicated that the addition of 150 and 200 U of CAT to the diet could significantly upregulate the mRNA expression of JAM2, MUR2, and occludin in the duodenum of yellow broilers in the early stage and the mRNA expression of JAM2 in the jejunum in the later stage. Adding 200 U of CAT to the diet prominently increased the mRNA expression of occludin in the jejunum, and JAM2 and occludin in the ilea in the early stage, and significantly raised the expression of MUR2 in the duodenum, MUR2, occludin, ZO-1 in the jejunum and JAM2, MUR2, and occludin in the ilea in the later stage. In this experiment, we verified the influence of catalase on the gene transcription level of tight junction protein and mucin. As for the influence on their protein expression level, our team will continue to conduct in-depth research.

## Conclusion

The addition of CAT in the diet can increase the body weight gain and reduce the feed:gain ratio of yellow broilers, improve the antioxidant capacity of the body and the activity of antioxidant enzymes in the body, increase the villus height and the ratio of villus height-to-crypt depth of the intestinal tract and reduce the crypt depth, enlarge the number of beneficial microorganisms in the intestine, and upregulate the mRNA expression levels of tight junction protein and mucin.

The results of this study and previous studies by our team showed that adding 200 U/kg of CAT to the diet of yellow broilers has the best effect.

## Data Availability Statement

The original contributions presented in the study are included in the article/supplementary material, further inquiries can be directed to the corresponding author/s.

## Ethics Statement

The animal study was reviewed and approved by the Animal Welfare Committee of Hunan Agricultural University.

## Author Contributions

MT conceived and designed the study, performed the experiments, and wrote the original paper. MT, JX, and KY analyzed the sequencing data and experimental results. YL corrected the manuscript. RF obtained the project funding, guided the experiment, and revised the manuscript. All authors have read and agreed to the published version of the manuscript. All authors contributed to the article and approved the submitted version.

## Funding

We greatly appreciate the funding support of the National Key R&D Program of China (2018YFD0501403).

## Conflict of Interest

YL was employed by the company Shanghai Menon Biotechnology Co., LTD. The remaining authors declare that the research was conducted in the absence of any commercial or financial relationships that could be construed as a potential conflict of interest.

## Publisher's Note

All claims expressed in this article are solely those of the authors and do not necessarily represent those of their affiliated organizations, or those of the publisher, the editors and the reviewers. Any product that may be evaluated in this article, or claim that may be made by its manufacturer, is not guaranteed or endorsed by the publisher.
